# The nexus of dynamic T cell states and immune checkpoint blockade therapy in the periphery and tumor microenvironment

**DOI:** 10.3389/fimmu.2023.1267918

**Published:** 2023-10-10

**Authors:** Hong Luo, Wenxiang Wang, Jia Mai, Rutie Yin, Xuyu Cai, Qintong Li

**Affiliations:** ^1^ Department of Obstetrics & Gynecology, Laboratory Medicine and Pediatrics, West China Second University Hospital, Key Laboratory of Birth Defects and Related Diseases of Women and Children, Ministry of Education, Development and Related Diseases of Women and Children Key Laboratory of Sichuan Province, Center of Growth, Metabolism and Aging, State Key Laboratory of Biotherapy and Collaborative Innovation Center of Biotherapy, Frontiers Science Center for Disease-related Molecular Network, West China Hospital, Sichuan University, Chengdu, Sichuan, China; ^2^ Xinxiang Central Hospital, The Fourth Clinical College of Xinxiang Medical University, Xinxiang, Henan, China; ^3^ Institute of Respiratory Health, Frontiers Science Center for Disease-related Molecular Network, West China Hospital, Sichuan University, Chengdu, Sichuan, China

**Keywords:** immunotherapy, peripheral blood, T cell, TCR repertoire, immune checkpoint blockade, PD-1, PD-L1, single-cell analysis

## Abstract

Immune checkpoint blockade (ICB) therapies, that is, using monoclonal antibodies to reinvigorate tumor-reactive, antigen-specific T cells from the inhibitory effects of CTLA-4, PD-1 and PD-L1 immune checkpoints, have revolutionized the therapeutic landscape of modern oncology. However, only a subset of patients can benefit from the ICB therapy. Biomarkers associated with ICB response, resistance and prognosis have been subjected to intensive research in the past decade. Early studies focused on the analysis of tumor specimens and their residing microenvironment. However, biopsies can be challenging to obtain in clinical practice, and do not reflect the dynamic changes of immunological parameters during the ICB therapy. Recent studies have investigated profiles of antigen-specific T cells derived from the peripheral compartment using multi-omics approaches. By tracking the clonotype and diversity of tumor-reactive T cell receptor repertoire, these studies collectively establish that *de novo* priming of antigen-specific T cells in peripheral blood occurs throughout the course of ICB, whereas preexisting T cells prior to ICB are exhausted to various degrees. Here, we review what is known about ICB-induced T cell phenotypic and functional changes in cancer patients both within the tumor microenvironment and in the peripheral compartment. A better understanding of parameters influencing the response to ICBs will provide rationales for developing novel diagnostics and combinatorial therapeutic strategies to maximize the clinical efficacies of ICB therapies.

## 1 Introduction

Cancer immunotherapies targeting immune checkpoint pathways have changed the therapeutical landscape of medical oncology. Immune checkpoint inhibitors, that is, antibodies blocking cytotoxic T-lymphocyte associated protein 4 (CTLA-4), programmed cell death 1 (PD-1) as well as its ligand PD-L1, have been approved by regulatory agencies around the world to treat multiple human cancer types (1). Beyond CTLA-4 and PD-1/L1, new therapeutical modalities targeting other immune checkpoints are also rapidly emerging. In the phase 3 study, Relatlimab (anti-LAG3, lymphocyte activation gene-3) in combination with Nivolumab (anti-PD-1) provided a greater progression-free survival (PFS) benefit than Nivolumab monotherapy in melanoma patients (2), leading to regulatory approval by USA FDA to treat unresectable or metastatic melanoma. T-cell immunoglobulin mucin-3 (TIM-3; encoded by HAVCR2) is a co-inhibitory receptor suppressing effector Th1 cell function (3). In several phase 1 and 2 studies, anti-TIM-3 antibodies, including TSR-022 (NCT02817633), Sabatolimab (NCT02608268), and LY3321367 (NCT03099109), were broadly safe and well tolerated (4–6). VISTA (V-domain Ig suppressor of T cell activation) is an immune checkpoint receptor expressed on tumor-infiltrating T lymphocytes (TILs) and myeloid cells, leading to the suppression of T cell activation, proliferation, and cytokine production (7). HMBD-002, an anti-VISTA monoclonal antibody, is currently under clinical evaluation in triple-negative breast cancer (TNBC) and non-small cell lung cancer (NSCLC) patients (NCT05082610). Icatolimab (anti-BTLA, B and T lymphocyte attenuator), is being tested in phase 1 trial to treat melanoma patients (NCT04137900). In parallel, reagents directly stimulating immune activators are being tested clinically. Vopratelimab, an agonist for ICOS (inducible co-stimulator), demonstrated a favorable safety profile in phase 2 study as monotherapy and in combination with Nivolumab in head and neck squamous cell carcinoma (HNSCC), NSCLC and TNBC patients (8). GWN323, an IgG1 monoclonal antibody (mAb) against GITR (glucocorticoid-induced TNFR-related protein) (NCT04021043), was well tolerated in patients with relapsed/refractory solid tumors (9, 10). CP-870893, an agonist for CD40 signaling pathway, exhibited encouraging anti-tumor activity patients with metastatic melanoma in phase 1 study (11). 4-1BB agonists (Urelumab and Utomilumab), and OX40 agonist (INCAGN01949) seem to display good safety and tolerability profiles (12–14). Some of these newly developed modulators of immune checkpoints and activators are likely to achieve clinical success in near future and empower the arsenal of cancer immunotherapies.

Biomarkers to predict the initial response to and outcome of immune checkpoint blockades (ICBs) are of high clinical relevance. PD-L1 expression was initially considered as a reasonable biomarker to predict the response to anti-PD-1/PD-L1 therapies. Clinical studies have shown that patients with higher intratumor PD-L1 levels are more likely to benefit from ICBs (15, 16). These studies lead to current clinical practice using the PD-L1 expression level as a diagnostic criterion to guide patient selection. However, this stratification method is suboptimal. Patients with PD-L1-negative tumors can sometimes respond to ICBs (16, 17), and conversely ovarian cancer patients with high PD-L1 expression fail to do so (18). In addition, PD-L1 expression levels show temporal and spatial variation during tumorigenesis and cancer treatment (19–22). These complications have prompted enormous interest in the research community to identify additional markers beyond the expression of PD-L1.

Early studies have focused on using tumor specimens to identify predictive markers, such as tumor mutation burden and infiltrating lymphocytes (23–27). However, there are several caveats and limitations when using biopsy to interpret the dynamic interactions between tumor and immune cells as well as to guide the ICB therapies. First, patients with metastatic diseases are usually not suitable for biopsy or surgical operation (28, 29). Secondly, it is likely that in many cases biopsies only reflect a portion of tumor mass due to sampling errors and tumor heterogeneity (30–32). Of note, current guidelines to select suitable patients for anti-PD-1 and anti-PD-L1 therapies are based on the percentage of PD-L1^+^ cells (33–36). Pathological assessments of tissues obtained from biopsy or surgery may be biased due to variations on the sampling technique and the heterogenous nature of advanced diseases (30–32, 37, 38). Biopsy techniques are known to affect the accuracy of assessment of PD-L1^+^ cells, thus affecting patient stratification (39–42). Constrained sampling space also limits the clinical extrapolation and generalizability of results derived from biopsies (43, 44). Thirdly, in most clinical scenarios, it is impossible to obtain consecutive biopsies during the course of ICB therapies. After initial treatment, longitudinal monitoring is often required for early detection of relapsed diseases in any antitumor regimens (45, 46). It will be difficult to assess PD-L1 expression because relapsed tumor specimens are usually inaccessible. Thus, the dynamic changes in immunological parameters during ICB therapy cannot be monitored to guide treatment options.

Recently, immunologic profiling and immunophenotyping of patient peripheral blood have attracted much attention as a means to comprehensively evaluate antitumor immunity. Cancer is a systemic disease in a sense that it constantly exchanges information with the immune system. Of note, several studies have demonstrated that the efficacy of ICB therapies mainly rely on the *de novo* priming of cytotoxic T cells from periphery, rather than the reinvigoration of pre-existing tumor infiltrating lymphocytes within tumor microenvironment (47, 48). Other studies have concluded that both peripheral and local expansion of antigen-specific T cells are critical (49–53). The differences between these studies may reflect different tumor types and disease stages. Nevertheless, they all highlight the importance of peripheral T cell response for ICB therapies to control tumor progression.

In this review, we summarize the current knowledge of ICB-induced T cell dynamics in the tumor microenvironment and the peripheral compartment. A better understanding of parameters influencing the response to ICBs will likely facilitate treatment decisions and improve cancer management.

## 2 Immune checkpoint blockades

**Figure 1:**
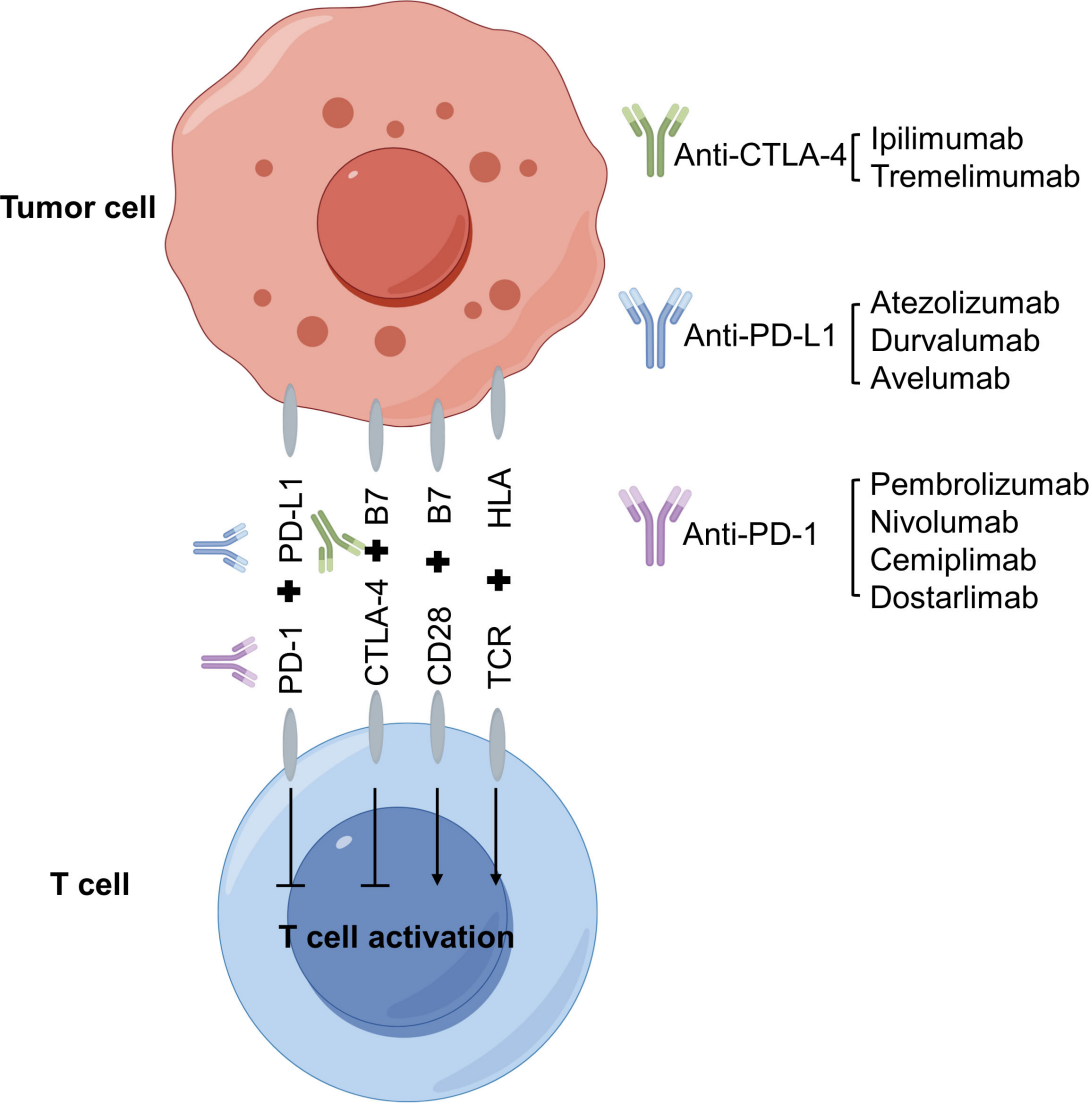
Mechanism of immune checkpoint blockade therapy. Immune checkpoints refer to the signaling pathways that dampen the activation of T cells by antigen presenting cells (APCs) or tumor cells. Immune checkpoint blockade (ICB) therapies use monoclonal antibodies to alleviate the inhibitory effects of immune checkpoints, including cytotoxic T-lymphocyte antigen 4 (CTLA-4) (Ipilimumab and Tremelimumab), PD-1 (Pembrolizumab, Nivolumab, Cemiplimab and Dostarlimab) and PD-L1 (Atezolizumab, Durvalumab and Avelumab). ICBs reinvigorate T cells via the activation of downstream signaling pathways such as MAPK and AKT-mTOR. Figure made by figdraw.

Antitumor immunity refers to the innate and adaptive immune responses to halt tumor initiation and progression. Successful antitumor immunity largely relies on the activation of cytotoxic T cell responses (**Figure 1**). To avoid overactivation of T cells, the immune system utilizes negative regulators, or immune checkpoints, such as PD-1, PD-L1 and CTLA-4 to dampen the cytotoxic effects of T cells. ICB therapies are based on therapeutic antibodies capable of blocking the inhibitory activities of CTLA-4, PD-1 and PD-L1, with the intention to prolong the antitumor effect of T cells (54–56). CTLA-4 is thought to regulate immune responses mainly at the early stage of T-cell activation by interrupting costimulatory effect of CD28 (57–60). Ipilimumab and Tremelimumab, therapeutic antibodies blocking CTLA-4, have shown durable control of tumor growth in some patients. However, overall survival (OS) rate is not significantly improved, and many patients do not respond to Ipilimumab and Tremelimumab such as in ovarian cancer (61) and melanoma (62, 63). These observations indicate that early stage of T cell response may be intact in most cancer patients. On the other hand, the interaction between PD-1 (mainly expressed on T cells) and PD-L1 (mainly expressed on tumor cells) was originally thought to inhibit effector T-cell activity within the tumor microenvironment (64–67). Several therapeutic antibodies targeting either PD-1 (Pembrolizumab, Nivolumab, Cemiplimab and Dostarlimab) or PD-L1 (Atezolizumab, Durvalumab and Avelumab) have been approved to treat malignancies such as those of lung, liver and breast (68–71). Durable control over tumor progression and improvement of OS have been achieved in responsive patients. Unfortunately, many patients do not respond to anti-PD-1 or anti-PD-L1 ICB therapies.

The clinical efficacy of ICB therapies varies among different tumor types. The first category is exemplified by melanoma and NSCLC. Many patients with these malignancies exhibit a high and durable response to ICB therapies. In other cancers such as those of colon and endometrium, most patients do not respond. However, a small subset of patients demonstrates exceptional responses due to high microsatellite instability and mismatch repair deficiency in the tumor (72, 73). One unifying mechanistic explanation for clinical benefits in melanoma, NSCLC and mismatch repair-deficient cancers is that these tumors usually contain high mutational burden, thus increasing the chance to generate neoantigens to elicit antitumor immunity (74). The third group, exemplified by ovarian cancers, is completely refractory to PD-1- or PD-L1-targeting ICBs, either in the setting of monotherapy or in combination with other anti-cancer drugs (18, 75, 76). Ovarian cancers are the most lethal gynecological cancer. The lack of clinical efficacy is surprising because tumor-infiltrating lymphocytes (TILs) are present in a substantial portion of ovarian cancer patients (77). Currently, it is unknown why ovarian cancers do not respond to ICBs. One potential explanation is that other checkpoint pathways such as TIM-3 are at play (78). Alternatively, lack of response to ICBs is consistent with the fact that most T cell infiltrates in ovarian tumors are bystanders reactive to viruses rather than tumor antigens (79).

## 3 T-cell receptor repertoire analysis and its application to dissect the mechanisms of ICB therapies

At the cellular level, tumor-derived antigens are presented by antigen presenting cells, such as dendritic cells, to T-cell receptor (TCR) expressed on the plasma membrane of T cells. Most TCRs in humans are dimers of an alpha (α) and a beta (β) chain. The amino acid sequences of α and β chains are highly diverse due to a random joining of variable (V), junctional (J) and diversity (D) gene fragments (80, 81). The α chain is generated by the recombination of VJ segments, and the β chain by VDJ recombination. The vast sequence diversity of α and β underlies the ability of TCR to recognize numerous tumor antigens to initiate antitumor immunity. Thus, the TCR repertoire, that is, the collection of nucleotide composition of α and β chains, is used to infer immunological status of patients.

Recent advances in bulk and single-cell RNA sequencing (RNA-seq) have enabled measurement of patient TCR repertoire (82–84). To determine TCR repertoire by bulk RNA-seq, RNA extracted from thousands of T cells is used to amplify VDJ gene segments of α and β chains, followed by next-generation sequencing. Bioinformatic tools are then used to annotate the sequence and abundance of each α and β chain. Bulk RNA-seq is low cost, but does not generate information on the paired α and β chain in each T cell. Although bioinformatic algorithms are available to infer paired α and β chains, their accuracy is moderate. The information of paired α and β chain in each T cell is important as the affinity of TCR to antigens is dictated by sequences of both α and β chains. Thus, single-cell RNA-seq of TCR repertoire (sc-TCR-seq) fulfills this gap albeit at a higher cost. Information generated from sc-TCR-seq can be used for downstream functional assays to identify and validate antigen-specific TCRs (85). The combination of bulk- and sc-TCR-seq allows comprehensive analysis and sensitive tracking of T-cell dynamics at the clonal level to understand ICB-induced antigen-specific T-cell responses.

Early studies suggested that TILs may be an independent predictor of patient survival. Originally, the mechanism-of-action of ICB therapies was believed to activate TILs for durable tumor control. It was assumed that ICBs activate pre-existing, intratumoral T cells (86). However, by mapping TCR repertoire, recent studies have collectively demonstrated that in responsive patients the peripheral compartment is the major source of T cells activated by ICB therapy, and a majority of pre-existing intratumoral T cells are terminally exhausted and refractory to ICB therapies. Depending on the disease stage, a small subcluster of pre-exhausted CD8^+^ T cells within TME, termed precursors of exhausted T cells or Tpex, may also contribute to ICBs (87). These observations imply that tumor compartment actively communicates with the peripheral immune system (88). ICB-induced clonal expansion of antigen-specific T cells may be indicated by a decrease in TCR diversity. Conversely, ICB-induced migration of T cells to the tumor may increase the diversity of TCR repertoire. Thus, diversity score can be calculated to allow statistical analysis of TCR clonality during ICB treatment as well as differential responses among patients.

## 4 Overview of the CD8^+^ T cell infiltrates in human tumor microenvironment

Tumor microenvironment (TME) is composed of intertwining cancer cells, immune cells, stromal cells, and extracellular matrix (89, 90). Recent studies using single-cell transcriptomic profiling and TCR repertoire sequencing have revealed that T cell infiltrates in TME are populated with tumor-reactive CD8^+^ T cells, but also with bystander CD8^+^ T cells recognizing viral antigens (91, 92). Bystander T cells do not contribute to the antitumor immunity, nor do they respond to ICBs (93). Different tumor types have various amount of bystander T cells. Notably, more than 80% TILs in ovarian tumors are bystanders (94). This may explain why they are refractory to ICB therapies.

**Figure 2:**
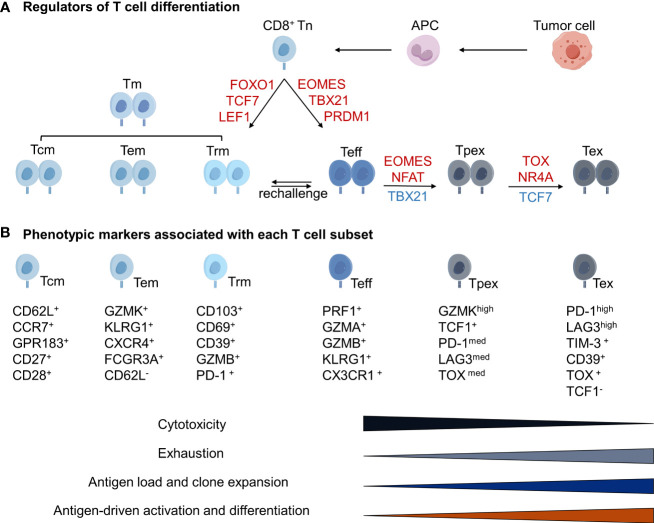
Diverse T cell states in human tumor and periphery compartments identified by single-cell analytic techniques. APCs promote resting native CD8^+^ T cells (Tn) to differentiate into cytotoxic, proliferative effector cells (Teff). Long-lived memory cells (Tm) are derived either from Teff or directly from Tn. Single-cell transcriptomic profiling studies further divide Tm into several subsets including central memory (Tcm), effector memory (Teff) and resident memory (Trm), based on differential gene expression profiles. Tm cells can differentiate into Teff cells when they are rechallenged by cognate antigens. In tumor microenvironment (TME), continuous exposure to tumor-derived antigens eventually induces Teff to enter a dysfunctional state, also known as exhausted state (Tex). Tex cells are characterized by increased expression of immune checkpoints and reduced cytotoxicity. Recent studies also identify a progenitor cellular state to Tex (Tpex), and have proposed that Tpex cells are the main cell population reinvigorated by ICB therapies. **(A)** Transcriptional regulators associated with each T cell state. Factors in red and blue denote increased and decreased expression, respectively. **(B)** Phenotypic markers associated with each T cell subset. FOXO1 (Gene ID: 2308); TCF7 (also known as TCF-1; Gene ID: 6932); LEF1 (Gene ID: 51176); EOMES (also known as TBR2; Gene ID: 8320); TBX21 (also known as T-bet; Gene ID: 30009); PRDM1 (also known as BLIMP1; Gene ID: 639); NFAT (Gene ID:32321); TOX (also known as TOX1; Gene ID: 9760); NR4A includes NR4A1 (Gene ID: 3164), NR4A2 (Gene ID: 4929) and NR4A3 (Gene ID: 8013). Figure made by figdraw.

Tumor-reactive cytotoxic CD8^+^ T cells are central to successful antitumor immunity in numerous pre-clinical mouse models, and thus the main focus in a majority of studies using patient specimens. Historically, CD8^+^ T cells are classified as naive, effector and memory T cell subsets, based on their differentiation status (**Figure 2**). Naive T cells (Tn) are resting T cells yet to be exposed to antigenic stimulation. Once stimulated by antigens, Tn cells develop into effector T cells (Teff) acquiring cytotoxic functionalities. A subset of Teff cells further converts into resting memory T cells (Tm) to prepare the host for future challenge of cognate antigens. CD8^+^ Tn cells can be identified by the expression of CC chemokine receptor 7 (CCR7), lymphoid enhancer binding factor 1 (LEF1), interleukin-7 receptor (IL7R), transcription factor 7 (TCF7; also known as T cell factor 1,TCF1) and selectin L (SELL) (95, 96). Teff cells are commonly identified by the expression of perforin 1 (PRF1), granzyme A (GZMA), GZMB and natural killer cell granule 7 (NKG7). They also express other cytotoxicity markers including CX3C chemokine receptor 1 (CX3CR1), killer cell lectin-like receptor subfamily G member 1 (KLRG1) and Fcγ receptor IIIA (FCGR3A) (49, 97). Recent characterization of TILs by high dimensional techniques have revealed several subclusters with potentially different functionalities within each classical classification. Different research groups have named these subclusters with different names. Their functional implications for ICBs warrant further investigation (49, 87, 95, 96).

The functional states of CD8^+^ Tm cell pool are the key to sustained tumor control. These long-lived cells can rapidly enter the mitotic division to generate a large quantity of Teff cells after restimulation by cognate antigens. CD8^+^ Tm cell pool consists of heterogeneous cell types differing in epigenetic, transcriptional and translational status (98). Two major subpopulations, CD8^+^ central memory T cells (Tcm) and CD8^+^ effector memory T cells (Tem), differ in several aspects. Tcm, abundant in the spleen, blood and lymph nodes, exhibits a high proliferative potential upon reactivation, usually identified by the expression of the lymphatic homing markers CD62L, CCR7, GPR183, CD27 and CD28 (51, 99). Tem, present in the spleen and blood, is highly cytotoxic upon reactivation, usually identified by the expression of GZMK, KLRG1, CXCR4 and FCGR3A but lack of CD62L (99, 100). In addition, recent studies have identified a third subcluster, tissue resident memory T cells (Trm), usually marked as CD103^+^CD69^+^ cell population. Trm is excluded from circulation and resides in tissues under steady-state conditions (53, 101, 102). Compared to Trm within adjacent normal tissues, Trm within tumors was found to increase the expression of dysfunctional markers such as TIM-3, PD-1, CTLA-4 and LAYN (51, 103), suggesting that they have experienced with tumor antigens.

Tumor-reactive, cytotoxic T cells eventually enter a terminally dysfunctional or exhausted cellular state (Tex) due to chronic and sustained stimulation by tumor antigens (49, 95, 96). Tex cells are characterized by increased expression of cell surface inhibitory receptors, PD-1, CTLA-4, LAG3 and TIM-3. Tex cells are also characterized by much reduced effector functions to produce inflammatory cytokines interleukin 2 (IL-2), tumor necrosis factor (TNF), and interferon gamma (IFNγ) (104–106). Additional regulators of Tex cells have been identified by recent studies, such as LAYN, CD39 and TOX (100, 107–112). Transcription factors TOX1 and TOX2 can cooperate with NR4A to induce the expression of multiple immune checkpoints including CTLA-4, PD-1 and TIGIT in CD8^+^ T cells (110), possibly by opening and increasing the chromatin accessibility of these genes for transcription (113). CD39 and CD73 are two ectonucleotidases that can convert extracellular ATP into adenosine, favoring immunosuppressive environment (114). Therapeutic monoclonal antibodies as well as small molecules targeting CD73 (115, 116), and therapeutic mAbs targeting CD39 are being evaluated by phase 1 studies (117). It will be of interest to see whether these reagents can activate Tex cells.

### 4.1 CD8^+^ T cell dysfunction is a progressive state within tumor microenvironment

Tex cells within tumors are composed of highly heterogenous cell populations, likely reflecting their natural history of responding to antigenic stimulation during tumorigenesis. Advanced, full-blown tumors contain highly exhausted, dysfunctional T cells due to persistent antigen stimulation, whereas tumors at early stages may contains T cells on the path to the terminally exhausted states (48, 118–120). In mice and humans, significant phenotypic diversity is observed in the intratumor T cell pool, reflected by various combinations and expression levels of inhibitory and co-stimulatory receptors, such as TIM-3, CTLA-4, CD39, 4-1BB and PD-1 (104, 121–123). A pre-dysfunctional cellular state (Tpex) has been identified in CD8^+^ T cells in various human cancers, characterized by higher expression of inhibitory receptor genes than naive and cytotoxic T cells, but lower than dysfunctional T cell population. These Tpex cells are defined by high expression of GZMK and moderate expression of genes such as PD-1 and LAG3 (96). In other studies, Tpex cells are also defined as GZMK^+^ and ZNF683^+^ cell population expressing low to moderate levels of inhibitory receptor genes (47, 49, 97, 99, 100). In contrast, terminally exhausted Tex cells express high levels of co-repressor molecules LAYN, PD-1, CTLA-4, TIM-3, LAG3 and TIGIT (47, 49, 95–97, 99, 100). These cells show much reduced ability to perform classical CD8^+^ T cell effector functions, diminished responsiveness to cytokine stimulation, and inability to form a memory T cell pool. These cellular characteristics are regulated by metabolic reprogramming and epigenetic modifications (124). Tex cells at early stage have stem cell-like and memory-like phenotypes, express low levels of effector transcripts, and have a strong proliferative capacity (125–128). In addition, they still express various effector molecules, such as IFN-γ and granzyme B. These observations suggest that Tex cells at early stage may still be functional, but also imply that they are true tumor-reactive T cells within tumor microenvironment (104, 105, 125, 129–131).

To our knowledge, there is no approved drugs to reverse the cellular state of Tex cells. In preclinical studies using animal models, recent findings have suggested that the manipulation of epigenetic and metabolic regulations might partially revive Tex cells (132–137). The efficacy of these interventions is yet to be evaluated by clinical trials. Because Tex cells within tumor bed express high levels of TIM-3 and LAG3, it is hypothesized that the combination of anti-PD-1/L1, anti-TIM-3 and anti-LAG3 treatment may reactivate Tex cells. This assumption is currently being tested in phase 1 studies (NCT03099109 and NCT02460224).

### 4.2 Characterization of CD8^+^ T cells in the tumor microenvironment during ICB therapies

ICBs hinge upon the reinvigoration of tumor-reactive cytotoxic immune cells, mainly CD8^+^ T cells (**Table 1**). In the context of PD-1 or PD-L1 blockade, the relevant CD8^+^ T populations within the tumor microenvironment (TME) are not exactly clear, but could be Tpex cells (87, 104, 125). There are highly heterogeneous Tex and Teff/Tm populations in tumors, and it is not fully understood which subpopulation is activated by the ICB therapies. Studies have suggested that PD-1 blockade promotes Tex cell (LAG3^+^TIM-3^+^GZMA^+^PD-1^+^) infiltration in TME (154). The exhausted CD8^+^ TILs could undergo clonal expansion after ICBs (47, 120, 139). In a study of NSCLC patients treated with Nivolumab or Pembrolizumab, high PD-1^+^ TILs were found to be related to significantly PFS and OS (140). In the neoadjuvant context of ICBs, tumor-specific CD8^+^ T cells isolated from non-major pathological response patients expressed high levels of genes associated with T cell dysfunction, such as TOX2, CTLA-4, TIM-3, and CD39. This observation confirms that terminally exhausted T cells cannot be reinvigorated by ICB therapies (138). Quantitative multiplex immunofluorescence analysis of melanoma tumors treated with ICBs revealed that the presence of some exhausted CD8^+^ T cells were in a progenitor state, termed Tpex, on the path to differentiate into Tex cells. Significantly, a higher percentage of Tpex cells was correlated with a longer duration of response to ICB therapy in melanoma patients (152). Similarly in NSCLC patients, Tpex cells, expressing low coinhibitory molecules (CD39^-^ TIM-3^-^ LAYN^-^) and high level of GZMK, increase in responsive tumors after treatment (50).

**Table 1:** Proposed biomarkers of clinical response to immune checkpoint blockade immunotherapy or prognosis based on immune cell populations.

Cancer type	Number of patients	Stage	Tissue	Drugs	Assessment method	Association with clinical outcome	Refs
Non-small cell lung cancer (NSCLC)	16	Stage I-IIIA	Tumor	anti-PD-1 (Nivolumab)	sc-RNA-seq	Tumor-specific CD8^+^ T cells isolated from patients in “non-major pathological response (non-MPR)” were often highly expressed with genes associated with T cell dysfunction (TOX2, CTLA-4, TIM-3, and CD39), while patients with MPR have higher expression of genes associated with memory (IL7R and TCF7) and effector function (GZMK).	(138)
NSCLC	52	Stage I-IV	Tumor	anti-PD-1	sc-RNA-seq, bulk RNA-seq, flow cytometry	Trm (TIM-3^+^PD-1^+^CD103^+^) enriched in responders to anti-PD-1 therapy and enriched for transcripts linked to cell proliferation (Ki67^+^) and cytotoxicity (GZMB^+^IFN-γ^+^TNF^+^IL-2^+^).	(51)
NSCLC	86	Stage I-VI	Tumor	anti-PD- 1 or anti-PD-L1	quantitative multiplex IF	CD8^+^Trm (CD103^+^CD49a^+^CD69^+^PD-1^+^CD39^+^)cells demonstrate enhanced proliferation and cytotoxicity toward autologous cancer cells. Higher density of CD8^+^Trm in immunotherapy-naive tumors is associated with improved outcomes and Trm density increases during immunotherapy in responders.	(52)
NSCLC	4	Metastatic	Tumor	anti-PD-1 (Pembrolizumab)	CITE-seq and TCR-seq	Neoantigen-reactive T cells are CD39^+^CXCL13^+^, and high-frequency clonotype.	(139)
NSCLC	164	Stage IV	Tumor	anti-PD-1 (Nivolumab or Pembrolizumab)	flow cytometry and IHC	High PD-1^+^ TILs related to significantly longer progression-free and overall survival.	(140)
NSCLC	36	Advanced	Tumor, blood	anti-PD-1 (Pembrolizumab)	paired sc-RNA-seq and TCR-seq	Tpex (CD39^-^TIM-3^-^LAYN^-^GZMK^high^) cells increased in responsive tumors after treatment. Texp were accumulated by local expansion in TME and replenished from peripheral T cells with both new and pre-existing clonotypes.	(50)
NSCLC	21	Stage I-III	Tumor, blood	anti-PD-1 (Nivolumab)	TCR-seq	The post-treatment tumor bed of patients with MPR was enriched with T cell clones that had peripherally expanded between weeks 2–4 after anti-PD-1, and the intratumoral space occupied by these clonotypes was inversely correlated with percent residual tumor.	(141)
NSCLC	27	Advanced	Blood	anti-PD-1	flow cytometry	PD-1^+^CD8^+^ T cells that proliferate in the peripheral blood of lung cancer patients receiving PD-1 therapy express CD28, CD38 and HLA-DR.	(142)
NSCLC	29	Stage I-IV	Blood	anti-PD(L)-1 (Pembrolizumab or Nivolumab or Atezolizumab)	flow cytometry	Effector-like CD8^+^ T cell (HLA-DR^+^CD38^+^Ki67^+^PD-1^+^CTLA-4^+^) increased in responders post-treatment.	(143)
NSCLC	79	Advanced	Blood	anti-PD-1 (Pembrolizumab or Nivolumab)	flow cytometry	A higher fold-change in the percentage of Ki67^+^ cells among PD-1^+^CD8^+^ T cells 7 days after the first dose significantly predicted DCB.	(144)
NSCLC	18	Stage I-IV	Blood	anti-PD-1 (Pembrolizumab or Nivolumab)	flow cytometry	An increase in the frequency of CD8^+^ T cells expressing TIM-3 during treatment was negatively correlated with response and clinical response and PFS.	(145)
NSCLC	60	Stage IV	Blood	anti-PD(L)-1 (Pembrolizumab, Nivolumab, Atezolizumab)	sc-RNA-seq and sc-TCR-seq	Expansion of CD8^+^ Tem cells (GZMK^+^PD-1^+^) with novel TCRs in PBMCs after ICI treatment could contribute to a better clinical outcome in patients with NSCLC.	(146)
NSCLC	21	Advanced	Blood	anti-PD(L)-1	TCR-seq	The diversity of the dominant TCR clone at baseline was correlated with DCB in patients receiving single-agent treatment.	(147)
NSCLC	40	Stage IIIB-IV	Blood	anti-PD(L)-1	flow cytometry, TCR-seq	Patients with high PD-1^+^ CD8^+^ TCR diversity before ICB treatment showed better response to ICB and PFS compared with patients with low diversity.	(148)
NSCLC	263	Recurrent and/or metastatic	Blood	anti-PD(L)-1	flow cytometry	Frequencies of CD8^+^ Tem cells (CCR7^−^CD45RA^−^) were higher in patients with non-HPD than those in patients with HPD. Elevated frequencies of tumor-reactive CD8^+^ Tex cells (PD-1^+^TIGIT^+^) predicted HPD.	(149)
NSCLC	12	Stage I-IIIA	Blood	anti-PD-1 (Nivolumab)	TCR-seq, exome sequencing and neoantigen	Neoantigen-specific T-cell clones from a primary tumor with a complete response on pathological assessment rapidly expanded in peripheral blood at 2 to 4 weeks after treatment; some of these clones were not detected before the administration of Nivolumab.	(150)
NSCLC	57	Stage II-IV	Blood	anti-PD-1 (Nivolumab)	flow cytometry, RNA-seq	Patients with high Tcm (CCR7^+^CD45RA^-^)/Teff(CCR7^-^CD45RA^+^) cell ratios had longer PFS.	(151)
Lung cancer	6	Advanced	Tumor/blood	anti-PD-L1	sc-RNA-seq and TCR-seq	Patients with effector-like T cell expansion respond best to anti-PD-L1 therapy. Expanded T cell clones in tumors derivate from peripherally expanded clones.	(48)
Melanoma	32	Metastatic	Tumor	anti-PD-1 or anti-PD-1 plus anti-CTLA-4	IF	Elevated frequencies of TCF7^+^CD8^+^ T cells in fixed tumor specimens predict positive outcome.	(49)
Melanoma	25	Stage III-IV	Tumor	anti-PD-1 and anti-CTLA-4 (Nivolumab, Ipilimumab)	quantitative multiplex IF	Patients with a higher percentage of Tpex cells (TCF7^+^PD-1^+^) experience a longer duration of response to ICB therapy.	(152)
Melanoma	53	Stage IV	Tumor	anti -PD-1 (Pembrolizumab)	sc-RNA-seq, flow cytometry	CD8^+^ Tem (CD45RO^+^CCR7^-^CD27^-^CD57^-^) is the major T-cell expanded in patients with a response to therapy.	(153)
Melanoma	3	Stage III-IV	Tumor/blood	anti-PD-1	paired sc-RNA and sc-TCR-seq	PD-1 blockade promotes Tex (LAG3^+^TIM-3^+^GZMA^+^PD-1^+^) clonotype infiltration in TME, which is rarely found in blood.	(154)
Melanoma	4	Stage III-IV	Tumor/blood	anti-PD-1	paired sc-RNA and sc-TCR-seq	Cell populations sharing TCR clones are more activated (CCL5^+^GZMB^+^KLRG1^+^KLRK1^+^CX3CR1^+^) in peripheral blood and more exhausted (PD-1^+^LAG3^+^CTLA-4^+^TIM-3^+^TIGIT^+^) in TME.	(155)
Melanoma	4	Stage III-IV	Tumor/blood	anti-PD-1 or radiation	sc-RNA-seq, sc-TCR-seq, bulk-TCR-seq	Highly expanded clonotypes in TME were distributed predominantly in Tex, which is rarely found in blood. The persistence of Tex-related TCR clonotypes in peripheral blood increased in patients with a poor response to ICB.	(120)
Melanoma	27	Stage III-IV	Tumor/blood	anti-PD-1 (Pembrolizumab)	flow cytometry, RNA-seq	Peripheral Tpex (TCF1^-^T-bet^high^Tox^med^) re-engaged some effector biology and increased upon PD-L1 blockade but ultimately converted into terminally exhausted subset in TME.	(156)
Melanoma	7	Advanced	Tumor/blood	surgery, chemotherapy and immunotherapy	exome and RNA-seq, neoantigen prediction,coculture	CD8^+^PD-1^+^ T cell populations had lymphocytes that targeted neoantigen, and the tumor-antigen specificities and TCR repertoires of the circulating and tumor-infiltrating CD8^+^PD-1^+^ cells appeared similar.	(157)
Melanoma	29	Metastatic	Tumor/blood	anti-PD-1 (Nivolumab) or anti-CTLA-4(Ipilimumab)	flow cytometry, IF, sc-RNA-seq	TCF7^+^PD-1^+^ TILs mediated the proliferative response to immunotherapy, generating both TCF7^+^PD-1^+^ and differentiated TCF7^-^PD-1^+^ cells. Human TCF7^+^ PD-1^+^ cells were detected among tumor-reactive CD8^+^ T cells in the blood and tumor.	(158)
Melanoma	40	Stage IV	Blood	anti-PD-1	mass cytometry	Responders have lower frequency of CD8^+^ naive T cells (CD45RO^−^CD62L^+^) at baseline and after the start of treatment. Increased activation (CTLA-4^+^PD-1^+^) in CD8^+^ T cells after the initiation of immunotherapy in responders.	(159)
Melanoma	27	Stage III- IV	Blood	anti-PD-1 (Pembrolizumab)	flow cytometry	CD8^+^ Tex cells (Ki67^+^PD-1^+^CTLA-4^+^) increased in responder at day 7 post-treatment.	(160)
Melanoma	29	Stage IV	Blood	anti-PD-1 (Pembrolizumab)	flow cytometry	CD8^+^ Tex cells (CTLA-4 ^+^2B4^+^PD-1^+^) that proliferate in the peripheral blood in patients receiving PD-1 therapy.	(161)
Melanoma	26	Stage III -IV	Blood	anti-PD-1 (Nivolumab)	flow cytometry, RNA-seq, TCR-seq, IFN-γ ELISPOT assays	The frequency of circulating PD-1^+^TIGIT^+^CD8^+^ T cells after 1 month of anti-PD-1 therapy was associated with clinical response and OS, exhibiting an activated phenotype (HLA-DR^+^CD38^+^).	(162)
Melanoma	50	Metastatic	Blood	anti-PD-1 (Pembrolizumab) or anti-PD-1 plus anti-CTLA-4 (Nivolumab plus Ipilimumab)	flow cytometry, TCR-seq	Expansion of CD8^+^ T cells (CD45RA^-^CD45RO^high^CD27^-^CCR7^-^) correlated with response. ICBs induce peripheral T cell repertoire rearrangements and recruitment of T cells with a broader TCR repertoire from the periphery to the tumors in responder.	(163)
Melanoma	27	Metastatic	Blood	anti-PD-1 (Pembrolizumab) or anti - PD-1 plus anti-CTLA-4 (Nivolumab plus Ipilimumab)	sc-RNA-seq and sc-TCR-seq	Effector CD8^+^ T cells showed increased sensitivity to ICB. Cytotoxic clones (IFNγ-correlated genes) demonstrated a propensity to persist after ICB treatment. CD8^+^ T cell cytotoxicity is associated with clinical response to ICB.	(164)
Melanoma	137	Stage IV	Blood	anti-CTLA-4 (Ipilimumab)	flow cytometry	High frequencies of CD8^+^ Tem cells at baseline correlated with longer OS and higher clinical response rates especially in PD-1^+^CD8^+^Tem. High baseline frequencies of late stage-differentiated CD8^+^ Tem cells were negatively associated with OS but did not correlate with clinical response.	(165)
Melanoma	55	Metastatic	Blood	anti-PD-1 (Pembrolizumab) or anti-PD-1 plus anti-CTLA-4 (Nivolumab plus Ipilimumab)	sc-RNA-seq and sc-TCR-seq	Responding patients have more dominant clones (those occupying >0.5% of repertoire) post-treatment than non-responding patients or controls, and these clones over-express genes implicated in cytotoxicity and characteristic of Tem cells including CCL4, GNLY, and NKG7.	(166)
Melanoma	12	Metastatic	Blood	anti-PD-1	RNA-seq, flow cytometry	Circulating CX3CR1^+^CD8^+^ T cells are increased in patients with metastatic melanoma who responded to ICB, and have effector memory phenotypes and cytolytic activity (GZMB and perforin)	(167)
Melanoma	43	Stage IV	Blood	anti-CTLA-4 (Ipilimumab)	flow cytometry	CD8^+^ Tem cell (CCR7^-^CD45RA^-^) frequencies at the end of treatment were higher in patients with clinical benefit and positively correlated with survival.	(168)
Melanoma	30	Metastatic	Blood	anti-CTLA-4 (Ipilimumab)	flow cytometry	Baseline levels of CD45RO^+^CD8^+^ Tmem cells correlate with response and longer survival with Ipilimumab, and express HLA-DR^+^CD25^−^ phenotype.	(169)
Melanoma	36	Stage IIIC-IV	Blood	anti-CTLA-4 (Ipilimumab)	flow cytometry	Increased EOMES^+^CD8^+^ T cell and GZMB^+^EOMES^+^CD8^+^ T cell (Teff) at 6 months were significantly associated with relapse. Low Ki67^+^EOMES^+^CD8^+^ T cells were associated with relapse at baseline.	(170)
Basal cell carcinoma (BCC)	11	Advanced or metastatic	Tumor	anti-PD-1 (Pembrolizumab or Cemiplimab)	paired sc-RNA-seq and TCR-seq	CD8^+^ Tex cells (TIM-3^+^TIGIT^+^) were clonally expanded and express markers of tumor-specificity (CD39^+^), and the majority of expanded clones were derived from novel clonotypes.	(47)
Squamous cell carcinoma (SCC)	4	Advanced or metastatic	Tumor	anti-PD-1 (Pembrolizumab or Cemiplimab)	paired sc-RNA-seq and TCR-seq	CD8^+^ Tex cells (TIM-3^+^TIGIT^+^) were clonally expanded and express markers of tumor-specificity (CD39^+^), and the majority of expanded clones were derived from novel clonotypes.	(47)
Merkel cell carcinoma (MCC)	15	Stage III -IV	Blood	anti-PD-1 (Pembrolizumab)	flow cytometry	The frequency of circulating PD-1^+^TIGIT^+^CD8^+^ T-cells after 1 month of anti-PD-1 therapy was associated with clinical response and OS, exhibiting an activated phenotype (HLA-DR^+^CD38^+^).	(162)
Triple-negative breast cancer (TNBC)	44	Early stage	Tumor	Treatment-naive or neoadjuvant chemotherapy	flow cytometry	CD8^+^ Trm (CD39^+^CD69^+^CD103^+^) were enriched in TNBC samples, correlating with OS of patients, and could be reinvigorated from exhaustion with the addition of ICB ex vivo.	(171)
Endometrial adenocarcinoma	3	Advanced	Tumor/blood	anti-PD-L1	sc-RNA-seq and TCR-seq	Patients with effector-like T cell expansion responded best to anti-PD-L1 therapy. Expanded T cell clones in tumors were derivated from peripherally expanded clones.	(48)
Colorectal adenocarcinoma	2	Advanced	Tumor/blood	anti-PD-L1	sc-RNA-seq and TCR-seq	Patients with effector-like T cell expansion responded best to anti-PD-L1 therapy. Expanded T cell clones in tumors were derivated from peripherally expanded clones.	(48)
Renal cell carcinoma (RCC)	3	Advanced	Tumor/blood	anti-PD-L1	sc-RNA-seq and TCR-seq	Patients with effector-like T cell expansion responded best to anti-PD-L1 therapy. Expanded T cell clones in tumors were derivated from peripherally expanded clones.	(48)
RCC	36	Stage IV	Tumor/blood	anti-PD-1 (Nivolumab) or anti-PD-1 plus anti-CTLA-4 (Nivolumab plus Ipilimumab)	flow cytometry, RNA-seq, TCR-seq	Highest increase in these HLA-DR^+^CD38^+^CD8^+^ T cells (Ki67^high^GZMB^high^CXCR3^high^) in peripheral blood after treatment had the best antitumor immune response and experienced clinical benefit. Responding patients had an influx of new TCR clonotypes to the HLA-DR^+^CD38^+^pool after ICB therapy.	(172)
RCC	7	Stage III-IV	Blood	anti-PD-1 (Pembrolizumab or Nivolumab)	flow cytometry	An increase in the frequency of CD8^+^ T cells expressing TIM-3 during treatment was negatively correlated with response and clinical response and PFS.	(145)
Head and neck squamous cell carcinomas (HNSCC)	30	Stage I-IV	Tumor/blood	anti-PD-1 (Nivolumab) or anti-PD-1 plus CTLA-4 (Nivolumab plus Ipilimumab)	sc-RNA-seq and TCR-seq	Treatment-expanded tumor T cell clones (CD103^+^ZNF683^+^GZMB^+^PD-1^+^CTLA-4^+^) in responding patients recognized cancer-specific antigen MAGEA1. The frequency of activated blood CD8^+^ T cells (CD38^+^HLA-DR^+^), notably pre-treatment PD-1^+^ KLRG1^-^ T cells, was strongly associated with intra-tumoral pathological response.	(53)
HNSCC	10	Stage I-IV	Blood	anti-PD-L1	mass cytometry	Tpex (PD-1^+^TCF7^-^CD69^-^) increased and expressed Ki67 in the blood following ICB.	(173)
HNSCC	41	Metastatic	Blood	anti-PD-1 (Nivolumab)	TCR-seq	Responder (CR and PR) had an increased TCR sequence diversity in their baseline samples. Patients within the same response group had shared TCR clones.	(174)
Thymic epithelial tumors	31	Advanced	Blood	anti-PD-1 (Pembrolizumab)	flow cytometry	A higher fold-change in the percentage of Ki67^+^ cells among PD-1^+^CD8^+^ T cells 7 days after the first dose significantly predicted DCB.	(144)
Esophageal squamous cell carcinoma	20	Advanced	Blood	anti-PD-1 (Nivolumab)	flow cytometry	TIM-3^+^in CD4^+^ and CD8^+^ T cells in CR/PR patients increased after the first cycle, but did not in SD/PD patients.	(175)
Gastrointestinal (GI) cancers	13	Stage III-IV	Blood	anti-PD-1 (Pembrolizumab)	sc-RNA-seq, flow cytometry	Responders’ CD8^+^ T cells showed greater up-regulation of the IFN-γ gene and IFN target genes (IRF1/2/7, STAT1/2, and IFN-stimulated genes). Responders had a higher frequency of cytotoxic differentiated CD8^+^ T cells than non-responders, both before and during treatment.	(176)
MSI-H gastric cancer	28	Metastatic	Tumor/blood	anti-PD-1 (Pembrolizumab)	sc-RNA-seq, flow cytometry, sequencing of whole exome and whole transcriptome	Responders demonstrated abundant preexisting TILs, a diverse pretreatment TCR repertoire, and a high proportion of stem-like exhausted cells in dysfunctional CD8^+^ TILs. An increase in PD-1^+^CD8^+^ T cells at baseline in periphery correlated with DCB.	(177)
Classical Hodgkin lymphomas (cHLs)	56	Relapsed	Blood	anti-PD-1 (Nivolumab)	TCR-seq, mass cytometry	Anti-PD-1 therapy was most effective in patients with a diverse baseline TCR repertoire and an associated expansion of reactive clones during treatment.	(178)

ICB, immune checkpoint blockade; ICIs, immune checkpoint inhibitors; IF, immunofluorescence; IHC, immunohistochemistry; sc-RNA-seq, single cell RNA sequencing; sc-TCR-seq, single cell T cell receptor sequencing; DCB, durable clinical benefit; PFS, progression-free survival; OS, overall survival; MPR, major pathological remissions; HPD, hyperprogressive disease; CR, complete remission; PR, partial remission; SD, stable disease; PD, progression disease; TIL, tumor-infiltrating lymphocyte.

Therefore, PD-1 inhibitors may reduce or revert the dysfunctional state of tumor-specific CD8^+^ T cells in TME. The extent that ICBs can reinvigorate Tex cells is influenced by several cell intrinsic features. The cellular state of Tex is determined by transcriptional and epigenetic factors (133, 179, 180). Several studies have demonstrated that PD-1 blocking antibodies can briefly revive Tex cells. However, this effect is not durable because these antibodies cannot reverse the epigenetic status of Tex cells (181–183). Nevertheless, a sustained response to the ICB therapies occur in some patients, indicating that Tex cells within TME may be in the progenitor rather than the fully differentiated state. However, other studies suggest that ICBs primarily promote the proliferation of a stem-like TILs subset, but not the reversal of T cell exhaustion programs. The majority of TME in stage IV metastatic melanoma patients who benefited from TIL-adoptive T cell therapy (ACT) had molecular characteristics of stem-like memory T cells, including the expression of TCF7, KLF2 and CD62L (184). Analysis in renal cancer TME revealed that a population of stem memory T cells with TCF7^+^TIM-3^-^CD28^+^ phenotype continued to proliferate and differentiate into tumor-killing effector T cells. Patients without such stem memory T cells in TME had an overall reduced T-cell infiltration and poor clinical prognosis (185). Similarly, in MSI-H gastric cancer, patients who responded to Pembrolizumab had a high proportion of stem-like exhausted cells in dysfunctional CD8^+^ TILs (177). Collectively, these studies suggest that different tumor stages, and thus the degrees of T cell exhaustion, is one of major determinants of ICB responsiveness.

The TCF7^+^PD-1^+^ stem memory T cell subpopulation was shown to be the primary source of T cells in mouse TME that could generate a sustained response to immunotherapeutic regimens such as ICBs and tumor vaccines. This subpopulation shares some transcriptomic similarity with Tm cells, but differs markedly at the epigenetic level (152). Other studies have shown that cells with effector-memory characteristics respond in ICB therapy (49, 153). In melanoma treated with Pembrolizumab, the CD8^+^ Tem subset (CD45RO^+^CCR7^-^CD27^-^CD57^-^) is the major expanded population in responsive patients (153). In another study, elevated frequencies of TCF7^+^ CD8^+^ T cells in melanoma specimens after ICBs were found to predict positive outcome (186). The molecular and phenotypic features, and the origin of this subpopulation of “tumor-specific Tm cells” in human TME are a subject of intensive research due to its relevance to ICB therapies (87, 103, 158, 186).

Many studies have indicated that Trm cells play a critical role in controlling tumor initiation and progression (51–53, 171, 187, 188). Trm cells mostly reside in tissues and do not recirculate in the bloodstream, even after reactivation by cognate antigens. RNA-seq analysis of excised NSCLC tumors shows that tissue-resident memory features are linked to the magnitude of cytotoxic T cell responses and better survival outcome (188). In NSCLC, Trm (TIM-3^+^PD-1^+^CD103^+^) cells expressing proliferation (Ki67^+^) and cytotoxicity (GZMB^+^IFN-γ^+^TNF^+^IL-2^+^) markers are enriched after anti-PD-1 therapy (51, 52). In TNBC, analysis by multicolor flow cytometry showed that Trm (CD39^+^CD69^+^CD103^+^) were enriched, correlated with OS, and could be reinvigorated by ICBs ex vivo (103, 171). In HNSCC, CD8^+^ T cells that underwent clonal expansion during ICB treatment expressed elevated tissue-resident memory (CD103^+^ZNF683^+^) and cytotoxicity programs (GZMB^+^PD-1^+^CTLA-4^+^) and show tumor-specific recognition (53).

## 5 Characterization of CD8^+^ T cells in peripheral blood during ICBs

**Figure 3:**
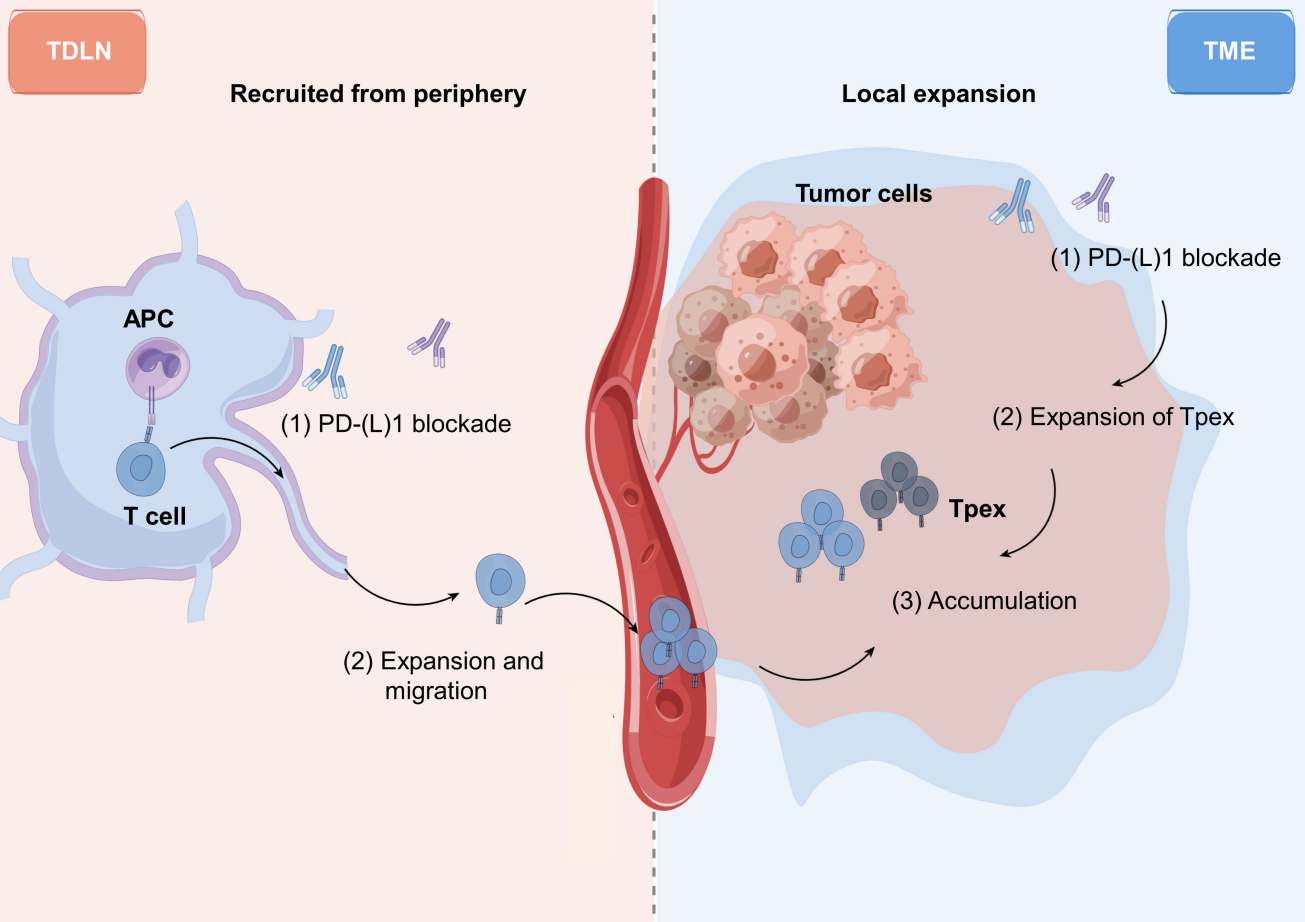
ICB therapies potentiate tumor-reactive CD8^+^ T cells in both periphery and TME. Single-cell transcriptomic and TCR repertoire analyses collectively suggest that the source of ICB-induced tumor-reactive T cells come from TME as well as peripheral lymph nodes. In ICB-responsive cancer patients, progenitor of exhausted T cells (Tpex) within TME, characterized by GZMK^high^, LAG3^med^ and PD-1^med^, are likely the cell population revived by ICB therapies. In responsive patients, peripheral tumor-reactive CD8^+^ T cells, containing different TCR sequences from Tpex cells, are also evident. These cells can enter TME to kill tumor cells. The relative contributions of peripheral tumor-reactive CD8^+^ T cells and Tpex in TME likely vary in individual patient. TDLN, tumor-draining lymph node; TME, tumor microenvironment. Figure made by figdraw.

In addition to TME, recent studies highlight the contribution of peripheral T cells during ICBs (**Figure 3**). In the peripheral compartment, lymph nodes are the primary location for antigen presentation cells to activate antigen-specific T cells (189, 190). Once activated, these T cells can migrate and infiltrate into the tumor to complement the effector population, emphasizing the importance of systemic immune responses (**Table 1**, **Figure 3**) (47, 48, 191).

The core of successful ICBs relies on the activation and proliferation of antigen-specific T cells. Paired sc-RNA and sc-TCR-seq analysis of melanoma patient specimens demonstrated that clonal T cells in periphery are in a more activated cellular state (CCL5^+^GZMB^+^KLRG1^+^KLRK1^+^CX3CR1^+^) than their counterparts in TME (PD-1^+^LAG3^+^CTLA-4^+^TIM-3^+^TIGIT^+^) (155). In the peripheral blood of NSCLC patients treated with anti-PD-1, CD28-positive CD8^+^ T cells were activated and proliferative (142). Circulating PD-1^+^ CD8^+^ T cells have been reported to be tumor-specific and share TCR sequences with PD-1^+^CD8^+^ TILs (157). Studies suggest that ICBs stimulate the proliferation of peripheral PD-1^+^CD8^+^ T cells (161, 173), and the frequency of this subset predicts the response to ICBs (143, 144, 160, 177). In melanoma patients treated with anti-PD-1 antibodies, peripheral CD8^+^ T cells were proliferative and showed co-expression of PD-1 and CTLA-4 at day 7 after treatment, but only in responsive patients (159–161). This subset of peripheral CD8^+^ T cells, expressing conventional activation markers (HLA-DR^+^CD38^+^) and proliferation marker (Ki67^+^), was associated with better response to ICB therapies in a variety of cancer types (53, 143, 144, 162, 172, 192).

One would expect that ICB-stimulated periphery T cells should have different clonotypes from those in TME. Indeed, TCR sequencing analysis revealed the emergence of new clonotypes in responsive patients, suggesting *de novo* priming of tumor-specific T cells (162, 172). Interestingly, the status of exhaustion marker TIM-3 on CD8^+^ T cells is associated with contrasting outcome in different cancer types. In NSCLC and renal cell carcinoma (RCC) patients treated with anti-PD-1, the frequency of TIM-3^+^CD8^+^ T cells was higher in non-responders (145). In contrast, in esophageal squamous cell carcinoma, higher frequency of TIM-3^+^CD8^+^ T cells was associated with responsive patients (175). This discrepancy may reflect the fact that inhibitory checkpoints are expressed in both progenitors of exhausted cells and highly exhausted cells. Thus, using single marker, such as TIM-3 or PD-1, may not be reliable to determine the functional state.

Peripheral CD8^+^ T cells with effector characteristics are closely associated with the immune response to ICBs. In gastrointestinal (GI) cancers, CD8^+^ T cells from responsive patients acquired greater cytotoxic capacity after immunotherapy (176). This CD8^+^ T cell cytotoxicity is also associated with clinical response to ICBs in melanoma (164). The expansion of a subset of peripheral CD8^+^ Teff cells (CD45RA^-^CD45RO^high^CD27^-^CCR7^-^) correlated with response in melanoma patients receiving anti-PD-1 and/or anti-CTLA-4 (163). In different types of cancers, greater expansion of effector-like T cells from periphery is highly correlated with the efficacy of ICBs (48). This response to ICBs is not limited to conventional Teff, but also occurs in Tpex that retain effector functions (50, 156). In NSCLC patients treated with Pembrolizumab, periphery-derived Tpex cells were increased in ICB responders (50). Similarly in melanoma, peripheral Tpex (TCF7^neg^T-bet^high^Tox^med^) had an “effector-like” profile and increased after treatment with Pembrolizumab. These “effector-like” CD8^+^ Tpex can be activated in the periphery by PD-1/PD-L1 blockade but not in the tumor (156). However, increased frequencies of CD8^+^ Teff (GZMB^+^EOMES^+^) at 6 months were associated with relapse in melanoma patients with Ipilimumab (170), suggesting that these cells might be terminally exhausted.

A higher percentage of specific subclusters of CD8^+^ Tm cells in peripheral blood at baseline is associated with better survival and enhanced clinical response (149, 151, 165, 169). NSCLC patients received Nivolumab with high Tcm (CCR7^+^CD45RA^-^)/Teff (CCR7^-^CD45RA^+^) ratios had longer PFS (151). High baseline frequencies of CD8^+^ Tem were correlated with longer OS and better clinical response in melanoma patients (165). The baseline levels of CD45RO^+^CD8^+^ Tm cells correlated with response rate and survival in melanoma patients treated with Ipilimumab (169). Amplification of CD8^+^ Tem in the peripheral blood during ICB therapies has also been reported to be associated with the response to ICBs. In stage IV NSCLC, ICB-induced expansion of peripheral CD8^+^ Tem cells (GZMK^+^PD-1^+^) with *de novo* primed TCRs are associated with favorable clinical outcome (146). In melanoma, responders have higher frequencies of Tem cell (CCR7^-^CD45RA^-^) after ICB treatment (168). In another melanoma study, increased CD8^+^ Tem expressing CX3CR1 was shown in patients responding to ICBs, and this subset has effector memory phenotypes and cytotoxic activity (167).

TCR diversity in peripheral blood is associated with clinical benefit in patients with ICB therapies (141, 150). Studies have shown that ICB therapies induce peripheral T cell repertoire rearrangements, and the recruitment of T cells from the periphery to the tumors in ICB responders in melanoma and NSCLC (163, 166). In a variety of tumor types, the effectiveness of ICBs correlates with the diversity in peripheral TCR repertoire either before or after the ICB therapy (147, 148, 174, 178). Indeed, various studies suggest that effective antitumor responses induced by ICB therapies may arise from *de novo* specificity generated in the periphery, and are therefore not affected by the immunosuppressive TME (47, 48, 166).

The role of CD4^+^ T cells in ICB-induced antitumor responses is far from clear. Only a few studies have mentioned the relationship between CD4^+^ T cells and response to ICBs. CD4^+^ Tem cell frequency in peripheral blood at baseline is associated with the responsiveness to ICB therapies (145, 193–196). Like CD8^+^ T cells, CD4^+^ T cells in responsive patients exhibited proliferative capacity, low expression of PD-1 and LAG3, and responded to PD-1 blockade *in vitro* and *in vivo* (195). In the context of melanoma, the frequency of peripheral CD4^+^ Tem (CD45RO^+^CD62L^-^) cells was higher in nonresponsive patients (159). The correlation of circulating Treg cells with ICBs is also unclear. Some studies suggested that responsive patients had a lower percentage of circulating Treg cells prior to anti-PD-1 than nonresponsive patients (194, 195), while others found no such correlation (159). Some studies even reported that a high percentage of circulating Treg cells before or after treatment was associated with better response (196–198). The discrepancy among these studies is unclear. To reconcile these findings, one could argue that higher percentage of circulating Treg cells may reflect a negative feedback mechanism to dampen tumor-reactive cytotoxic T cells during tumorigenesis.

Peripheral T cell analysis offers the advantage to monitor dynamic changes during the course of ICB therapies. However, there are also several limitations. The peripheral TCR repertoire is orders of magnitude higher than the TCR repertoire within the tumor environment, and dynamically influenced by environmental factors such as diet and infections. In contrast, the TCR repertoire within the tumor environment is relatively stable and simple, composed of limited TCRs reactive to tumor antigens. Thus, it is sometimes difficult to draw conclusions solely based on the analysis of the peripheral T cells. In fact, high levels of TIM-3^+^ CD8^+^ subset in the periphery are associated with both favorable and poor patient responses (145, 175).

Collectively, these studies highlight the positive association between the reinvigoration of tumor-reactive cells in the periphery and the responsiveness to anti-PD-1/PD-L1 therapies in cancer patients. Because the periphery is not constantly in contact with tumor cells, reinvigorated periphery T cells should be tumor-reactive and not in the exhausted state. In addition, ICBs are usually applied after the cytoreductive surgery, and the immunosuppressive tumor environment is alleviated. Thus, the combination of active T cell state and less suppressive microenvironment may allow reinvigorated periphery T cells to eliminate residual tumor cells after debulking surgery. However, these newly activated T cells will still succumb to mechanisms of primary and required resistance as their counterparts in the tumor bed, such as reduced antigen presentation due to loss of HLA molecules and attenuated interferon signaling pathways (199). Lastly, the exact nature of reinvigorated periphery T cells warrants further investigation. Tpex cells within the tumor bed are likely the primary target of ICBs. It is clear ICBs also activate tumor-reactive T cells in the periphery, and these cells can have the same TCRs as Tpex cells within tumor. However, shared coloniality does not necessarily mean common origin. Considering the enormous diversity of periphery TCR repertoire, it is possible that ICB-activated periphery T cells are *de novo* primed rather than from tumor Tpex cells. In fact, peripheral T cells from healthy donors can react to tumor associated antigens or neoantigens derived from cancer patients (200). In this case, enhanced priming of T cells, such as by CD40 signaling agonists and dendritic cell vaccines, will likely synergize with ICBs (201). Indeed, ICBs can enhance the ability of dendritic cells to active antigen-specific T cells to boost anti-tumor immunity (202–204).

## 6 Conclusions and perspectives

ICB therapy was thought to activate preexisting, tumor-infiltrating CD8^+^ T cells. Recent advances in single-cell analytic techniques have enabled the reassessment of this assumption. Single-cell transcriptomic and TCR analyses allow the deduction of various T cell states with distinct functionalities. They also facilitate the identification of tumor-reactive T cell clones during tumorigenesis and through the course of ICB therapy. Beyond the tumor-infiltrating CD8^+^ T cells, the contribution of periphery tumor-reactive T cells are now appreciated as an important determinant of the effectiveness of ICBs. Thus, monitoring periphery TCR repertoire and T cell states can be an effective approach to assess the response to ICB therapies. The understanding of parameters influencing ICB response would ultimately provide rationales to improve ICB strategies. Patients with higher percentage of CD8^+^ Tem and Tpex subsets in either their tumor biopsies or peripheral blood will likely benefit from anti-PD-1 or anti-PD-L1 treatment. Retrospective analyses of current studies may provide baselines of these subsets for future clinical trials to stratify patient population beyond PD-L1^+^ cells. On the other hand, a common thread of the Tex subset among different tumor type is that these cells express high levels of PD-1, LAG3, TIM-3 and CD39. Thus, the combination of anti-PD-1/L1 with anti-LAG3 and/or anti-TIM-3 monoclonal antibodies are expected to achieve higher clinical efficacy (205, 206). In addition, antibody-drug-conjugates based on anti-CD39 monoclonal antibody are being developed, providing additional means to drug Tex cells (207). Ultimately, a better understanding of molecular characteristics of tumor-reactive T cells, either in TME or derived from the periphery, may help develop novel strategies to improve the response rate and the duration of ICBs.

ICBs have achieved remarkable therapeutic success in a variety of human cancers. Nevertheless, a large portion of cancer patients have yet to benefit from ICB therapies. Recent investigations have shed light on the-mechanism-of-action of ICBs in patients. Building on these discoveries, several research avenues are worth pursuing. From a technical and practical perspective, systematic cross-platform validation and integration of large-scale datasets from patients is needed to harmonize key findings. For example, the field needs to reach a consensus on the phenotypic markers for various T cell subsets in order to better define and compare patient samples at different disease stages. It is still unclear whether the cellular state of Tpex cells, the key target of ICBs, will change in the course of tumor initiation and progression. Single-cell sequencing technology is instrumental to elucidate immune cell compositions and states in basic research, but difficult to apply in clinical settings. Future studies are needed to translate findings from single-cell sequencing studies into clinically actionable parameters. Perhaps most importantly, can we rely on information on the composition of T cell subsets to guide future clinical trial designs? New therapeutical modalities targeting additional immune checkpoints and activators beyond PD-1/L1 and CTLA-4 are rapidly emerging. Stratification of patients based on the composition and functional states of T cells would certainly be advantageous in clinical trials. To achieve this goal, major efforts are needed to develop clinically actionable parameters to define each T cell subset.

## Author contributions

HL: Writing – original draft. WW: Writing – original draft. JM: Writing – original draft. RY: Writing – review & editing. XC: Writing – review & editing. QL: Writing – review & editing.
